# Prognostic value of DNA repair genes based on stratification of glioblastomas

**DOI:** 10.18632/oncotarget.17452

**Published:** 2017-04-27

**Authors:** Sun Kun, Qiwen Duan, Gang Liu, Jing-Min Lu

**Affiliations:** ^1^ Department of Neurosurgery, Huai’an First People's Hospital, Nanjing Medical University, Huai’an, China; ^2^ Department of Oncology, Taihe Hospital, Hubei University of Medicine, Hubei, China; ^3^ Department of Orthopedics, Huai’an First People's Hospital, Nanjing Medical University, Huai’an, China; ^4^ Department of Neurology, The Affiliated Huai’an Hospital of Xuzhou Medical University and The Second People's Hospital of Huai’an, Huai’an, China

**Keywords:** glioblastoma multiforme, COX, Kaplan-Meier, prognosis

## Abstract

Abnormal expression of DNA repair genes is frequently associated with cancerogenesis of many tumors, however, the role DNA repair genes play in the progression of glioblastoma remains unclear. In this study, taking advantage of large scale of RNA-seq data, as well as clinical data, the function and prognosis value of key DNA repair genes in glioblastoma were analyzed by systematically bioinformatic approaches. Clustering was performed to screen potentially abnormal DNA repair genes related to the prognosis of glioblastoma, followed by unsupervised clustering to identify molecular subtypes of glioblastomas. Characteristics and prognosis differences were analyzed among these molecular subtypes, and modular driver genes in molecular subtypes were identified based on changes in expression correlation. Multifactor Cox proportional hazard analysis was used to find the independent prognostic factor. A total of 15 key genes, which were significantly related to prognosis, were identified and four molecular subtypes of disease were obtained through unsupervised clustering, based on these 15 genes. By analyzing the clinical features of these 4 molecular subtypes, Cluster 4 was found to be different from others in terms of age and prognosis level. A total of 5 key DNA repair genes, CDK7, DDB2, RNH1, RFC2 and FAH, were screened to be significantly related to the prognosis of glioblastomas (*p* = 9.74e^−05^). In summary, the DNA repair genes which can predict the prognosis of patients with Glioblastoma multiforme (GBM) were identified and validated. The expression level of DNA repair genes shows the potential of predicting the prognosis and therapy design in targeting GBM.

## INTRODUCTION

According to the World Health Organization classification of tumors of the central nervous system, Glioblastoma multiforme (GBM) is the most common and lethal type of brain tumor [1]. It is exhibited by infiltrating neighboring tissue and shape-shifting without typical scope [2]. This tumor is highly invasive and is often combined with healthy brain tissue, which leads to a dismal prognosis after surgical resection [2]. Generally, the median survival time for newly diagnosed patients is within 2 years after standard therapy, which is surgical resection followed by adjuvant radiation therapy [3]. The satellite tumor cells which have spread from the primary tumor usually escape from treatment and lead to tumor recurrence [2].

The major function of DNA repair genes is responding to DNA damage that is induced in cells and through external environmental factors [4]. Mutation of these DNA repair genes could lead to defects or limitation of DNA repair abilities. This would further cause accumulation of DNA damage *in vivo* which increases the risk of canceration [5, 6]. Cancer tissues over-express DNA repair genes and therefore develop greater DNA repair capacity than normal tissue, resulting in therapy resistance [5]. In recent years, it has been proven that the occurrence of Glioma is closely related to the abnormal gene expression or abnormal protein structure of oncogenes and cancer suppressor genes [7]. Therefore, the application of microarrays plays an important role for glioma diagnosis, treatment and prevention. For instance, exploring the DNA damage signals and DNA repair process would help researchers find a new way of discussing pathogenesis of glioma, which could indicate the growth activity of tumor tissue and guide the clinical treatment and prognosis.

In this present study, a total of 539 glioblastoma tumor patient samples were selected and analyzed, from the Cancer Genome Atlas (TCGA) database, to find the relationship between the expression patterns of GBM associated DNA repair genes and different prognoses. Different molecular subtypes were built for further identification of the correlation between disease prognosis and different driver gene expression. Finally, a total 5 driver genes were found to be highly predictive of cancer recurrence and recurrence-free survival of GBM patients.

## RESULT

### Data download and preprocessing

**Figure 1:**
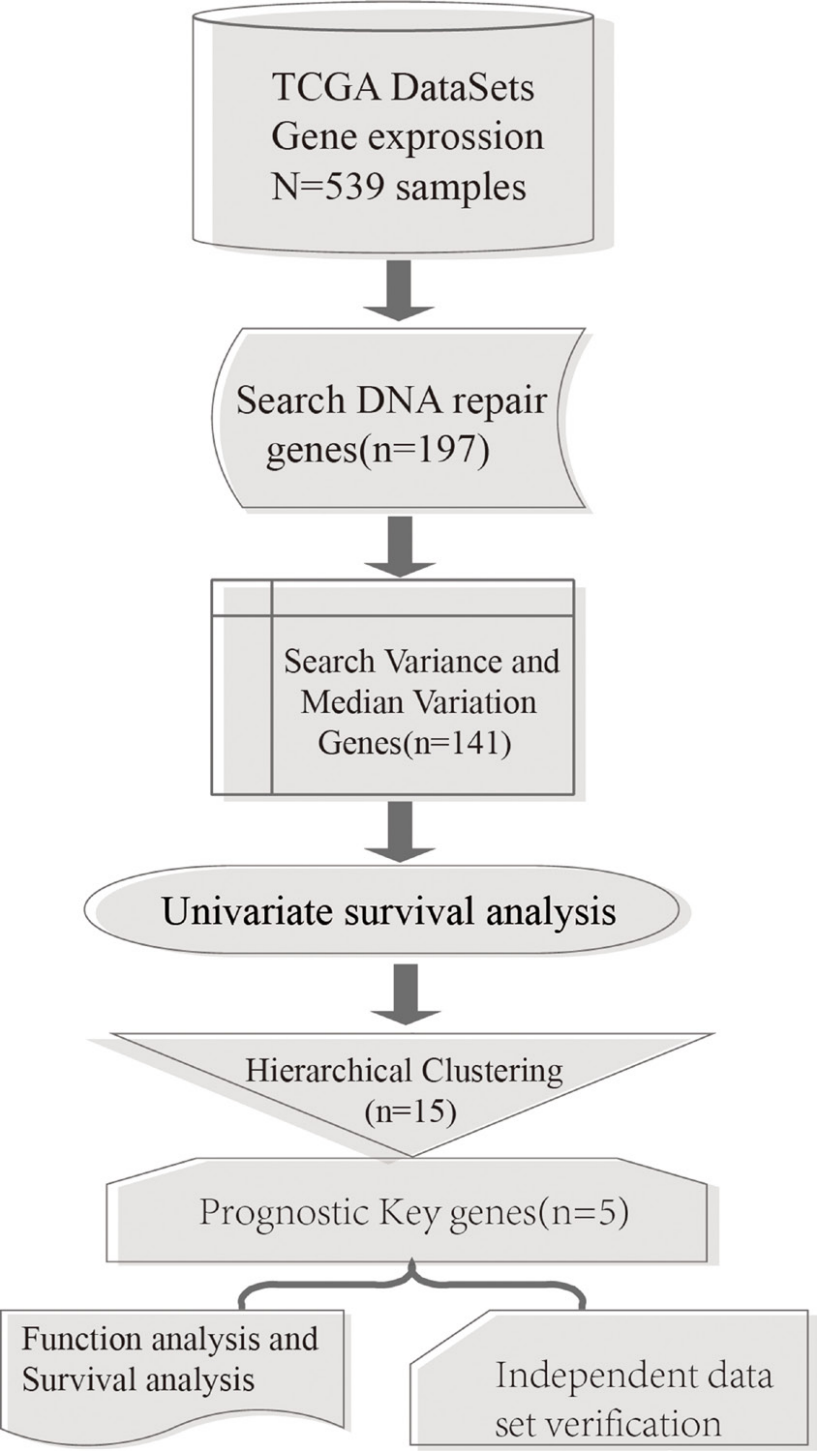
Process model flow chart

A total of 539 glioma samples which included 12042 gene expression values were obtained from TCGA database [8]. Among these, there were 498 primary glioma samples. A total of 194 expressed DNA repair genes were selected from these primary glioma samples. The process flow chart model is shown in Figure 1.

### Screening of potentially changed DNA repair genes

Of the 194 DNA repair genes, a total of 141 genes which reached statistical significance, above 20% of the genetic variance and the top 20% genetic median, were selected for further analysis. Then, 19 genes which had significant influence on prognosis were identified through single factor survival analysis [9]. In total, 15 genes had a variance > 0.7 and were selected as key DNA repair genes which significantly influence prognoses. The genes are shown in Table 1.

**Table 1:** DNA repair genes that influence prognosis significantly

Gene Symbol	Cox *p*-Values	Vars
CDK7	0.0210479144	0.8164490
RNASEH1	0.0063070069	0.7106625
MBD4	0.0444754216	1.0308578
MDC1	0.0106336900	0.7801713
FANCF	0.0390233510	0.9225329
FAH	0.0071272116	1.7431487
TOPBP1	0.0417646122	0.7349619
APEX1	0.0139679366	1.2269698
PMS2	0.0023484705	1.0100961
RFC2	0.0009096469	0.7259140
DDB2	0.0108868997	1.4985987
RNH1	0.0440518636	1.2254437
PARP2	0.0052141946	0.9035967
DCLRE1C	0.0253354384	1.1102217
DCLRE1A	0.0493212752	0.7516665

### Molecular subtypes construction

**Figure 2:**
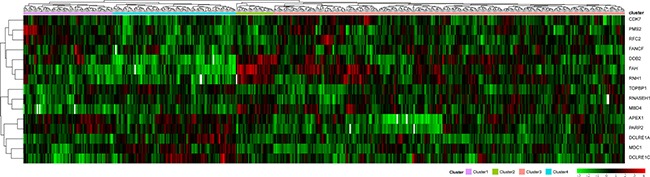
Clustering analysis of DNA repair genes which significantly influence prognosis The horizontal axis represents sample, using Euclidean distance to calculate distance; the vertical axis stands for genes, using Pearson correlation coefficient to calculate distance, horizontal axis divides the sample into 4 parts which are cluster 1, cluster 2, cluster 3 and cluster 4. The color in red represents level.

Unsupervised clustering was performed on the 15 DNA repair genes that were determined to significantly influence prognosis, shown in Figure 2. According to this figure, it can be seen that these 15 genes were divided into four groups. It can be seen that the number of samples were quite different between the 4 subtypes. Cluster 3 hold the largest amount of samples in the four clusters. The reason for the different sample sizes is that these 15 genes were screened out through simple factor analysis and the 4 clusters were clustered by these 15 selected genes. The number of samples in each cluster is random. Among each group, each gene showed significant differences in gene expression level. For example, in cluster 4, the expression levels of APEX1 (Apyrimidinic Endodeoxyribonuclease 1), PARP2 (Poly (ADP-Ribose) Polymerase 2), DCLRE1A (DNA Cross-Link Repair 1A), MDC1 (Mediator of DNA Damage Checkpoint 1), and DCLRE1C (DNA Cross-Link Repair 1C) were higher compared to the other clusters. In cluster 2, the expression levels of DDB2 (Damage Specific DNA Binding Protein 2), FAH (Fumarylacetoacetate Hydrolase) and RNH1 (Ribonuclease/Angiogenin Inhibitor 1) were higher than the other clusters.

### Clinical characteristics analysis of four molecular subtypes

**Figure 3:**
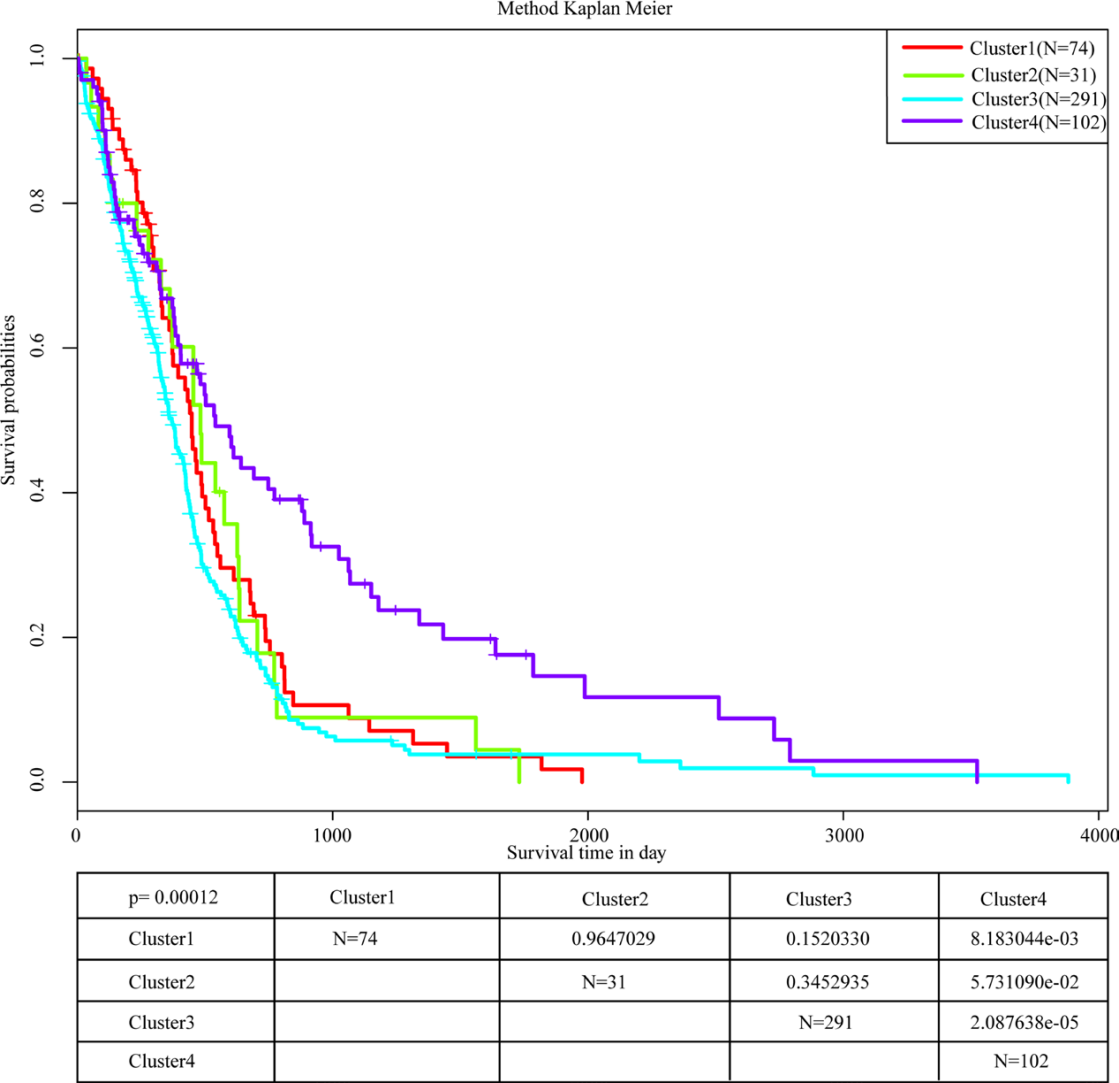
Prognostic analysis of 4 molecular subtypes The most significant difference occurs between cluster 3 and cluster 4, and the cluster 4 survival rate of 5 years is significantly higher than the rate of other clusters.

All samples from the four molecular subtypes were analyzed for prognostic outcome using the Kaplan-Meier [9] single factor survival analysis, shown in Figure 3. It can be seen that the prognosis of cluster 4 is significantly better than the other three clusters. The most significant difference occurred between cluster 3 and cluster 4, which was 0.0002451. This indicates that these 15 DNA repair genes could significantly distinguish different prognostic outcomes of different samples. The analysis of age distribution of the four clusters are shown in Table 2. Based on this analysis, the age distribution of cluster 4 was significantly different from the other three clusters, especially cluster 3 (0.0002451). This suggests that the positive prognosis of cluster 4 could be age-related.

**Table 2:** Age distribution and age difference test of 4 subtypes (mann-whitney *U* test)

Min-Median-Max	Cluster1	Cluster2	Cluster3	Cluster4
Cluster1	14-60-86	0.5545	0.7009	0.01421
Cluster2	0.5545	30-61-83	0.5777	0.03748
Cluster3	0.7009	0.5777	10-60-89	0.0002451
Cluster4	0.01421	0.03748	0.0002451	14-54-85

### Correlation analysis of expression of 15 DNA repair genes in 4 molecular subtypes

**Figure 4:**
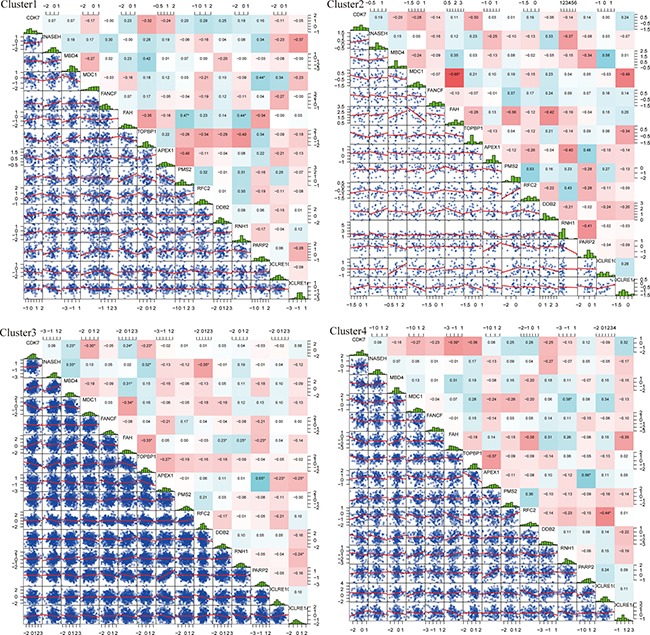
Expressed distribution and correlation of 4 subtypes genes Top matrix is correlation coefficient and bottom matrix is scatter diagram of gene expression level. Diagonal line is distribution of gene expression level.

We observed that 15 DNA repair genes from the four molecular subtypes showed differences in their individual expression correlation. Pearson Correlation Coefficient [10] was performed in order to determine the expressed correlation of these genes across all four molecular subtypes. The results are shown in Figure 4. The overall correlation between these 15 genes was small, which indicated that these genes likely play independent roles in biological pathways. Compared to the correlation of individual genes in different subtypes, it can be seen that the correlation of several genes changed significantly. For example, the correlation coefficient of CDK7 (Cyclin-Dependent Kinase 7) and FAH in cluster 1 was 0.23, while in cluster 4, the correlation coefficient was significantly different and decreased to -0.39. This is also exemplified with the MDC1 and FAH genes. In cluster 2, the correlation coefficient was -0.65 whereas in cluster 4 it was 0.07. These examples indicated that the same genes generate different regulated functions across different molecular subtypes.

**Figure 5:**
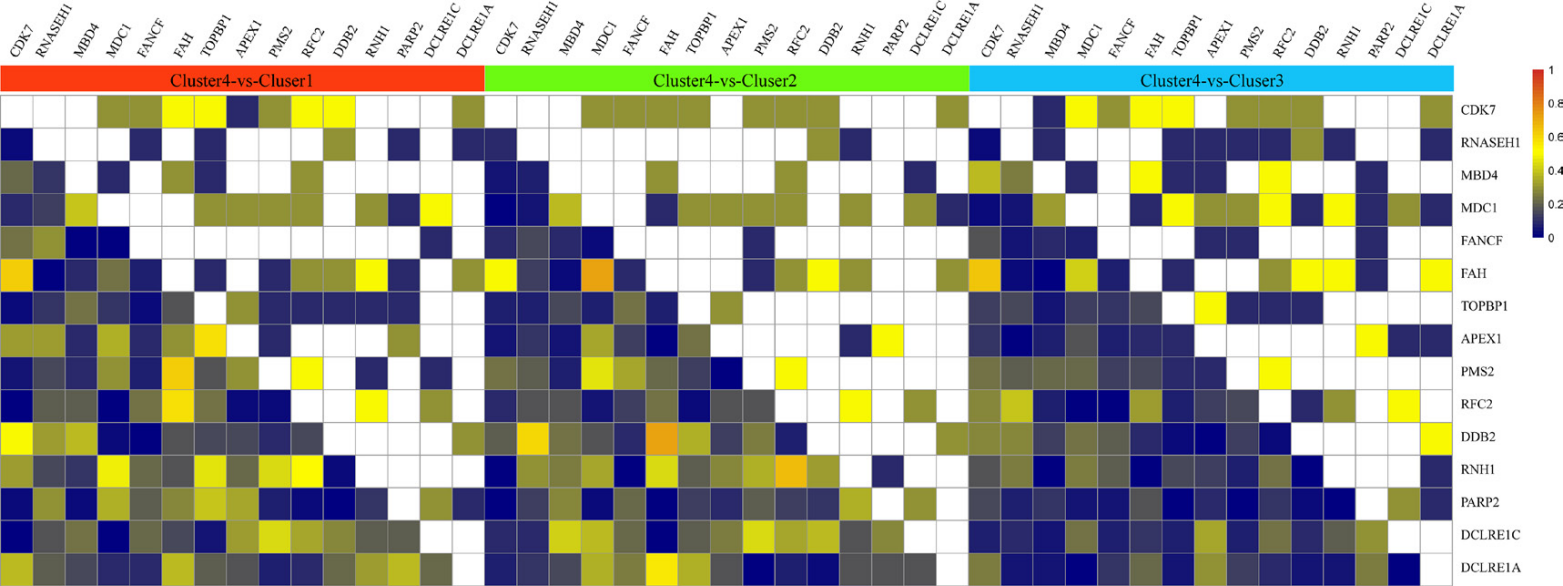
Correlation coefficient of cluster 4 compared with correlation coefficient matrix of the other 3 clusters The upper matrix stands for the significance of correlation coefficient. White color represents non-significant, yellow color represents significant correlation both in cluster 4 and the other clusters. The color from army green to dark blue means that compared with the other cluster, genes in cluster 4 show significant correlation, the darker the color is, the higher the significant was. The bottom matrix is the absolute value of correlation coefficient difference between cluster 1 and the other 3 clusters. The white square on diagonal line have no meaning.

In order to identify the differences between cluster 4 and the other three clusters, the correlation coefficient of cluster 4 was compared to the correlation coefficient matrix of the other three clusters. The results are shown in Figure 5. From this figure, it can be seen that comparing cluster 4 to cluster 1, CDK7 and FAH showed significant correlations in both clusters. However, these 2 genes were found to have significant positive correlation in cluster 1 whilst negative correlation in cluster 4 was observed. Genes RNH1 and RFC showed significant correlation in both cluster 1 and cluster 4, however, in cluster 1 this correlation was positive, while in cluster 4 the correlation was negative.

Comparing the gene correlation coefficient between cluster 2 and cluster 4, DDB2 and FAH had significant correlation in both clusters, which was negative in cluster 2, and positive in cluster 4. RFC2 and RNH1 showed positive correlation in cluster 2 and negative correlation in cluster 4.

Comparison of cluster 3 and cluster 4 showed that CDK7 and FAH were significantly and positively correlated in cluster 3, and negatively correlated in cluster 4.

In summary, alteration of the expression model of DNA repair genes could be the reason for different clinical survival rates between cluster 4 and the other three clusters. Among them, CDK7, DDB2, RNH1, RFC2 and FAH could act as potential driver genes.

### Functional enrichment analysis of drive genes

Functional enrichment analysis of driver genes from the four subtypes was performed through R package, clusterProfiler [11]. The results are shown in Table 3. It can be seen that genes CDK7, DDB2 and RFC2 were significantly enriched in 2 biological pathways, global genome and nucleotide-excision repair, as well as 3 Molecular Function which were mainly related to ATPase activity. In addition, DNA repair related biological pathways were enriched by the 5 genes.

**Table 3:** The significantly enriched GO terms of these 5 driver genes

	ID	Description	*q*-value	Gene ID
GO_BP	GO:0070911	global genome nucleotide-excision repair	2.37E-05	CDK7/DDB2/RFC2
	GO:0006289	nucleotide-excision repair	7.89E-05	CDK7/DDB2/RFC2
	GO:0033683	nucleotide-excision repair, DNA incision	0.000859	DDB2/RFC2
	GO:0006283	transcription-coupled nucleotide-excision repair	0.002274	CDK7/RFC2
	GO:0090305	nucleic acid phosphodiester bond hydrolysis	0.011614	DDB2/RFC2
	GO:0019439	aromatic compound catabolic process	0.013894	RNH1/FAH
	GO:1901361	organic cyclic compound catabolic process	0.014484	RNH1/FAH
GO_MF	GO:0008094	DNA-dependent ATPase activity	0.001207	CDK7/RFC2
	GO:0042623	ATPase activity, coupled	0.002934	CDK7/RFC2
	GO:0016887	ATPase activity	0.004229	CDK7/RFC2
KEGG	hsa03420	Nucleotide excision repair	3.45E-06	CDK7/RFC2/DDB2

### Multivariate survival analysis

To further explore the influence of these 5 driver genes on prognosis outcome, COX multivariate survival analysis was utilized. Likelihood ratio was used for analyzing the prognosis significance. The results were shown in Figure 5A and were found to be significant, *p* = 9.74e-05, which indicated that all 5 genes showed significant influence on prognosis.

### Independent data set validation

**Figure 6:**
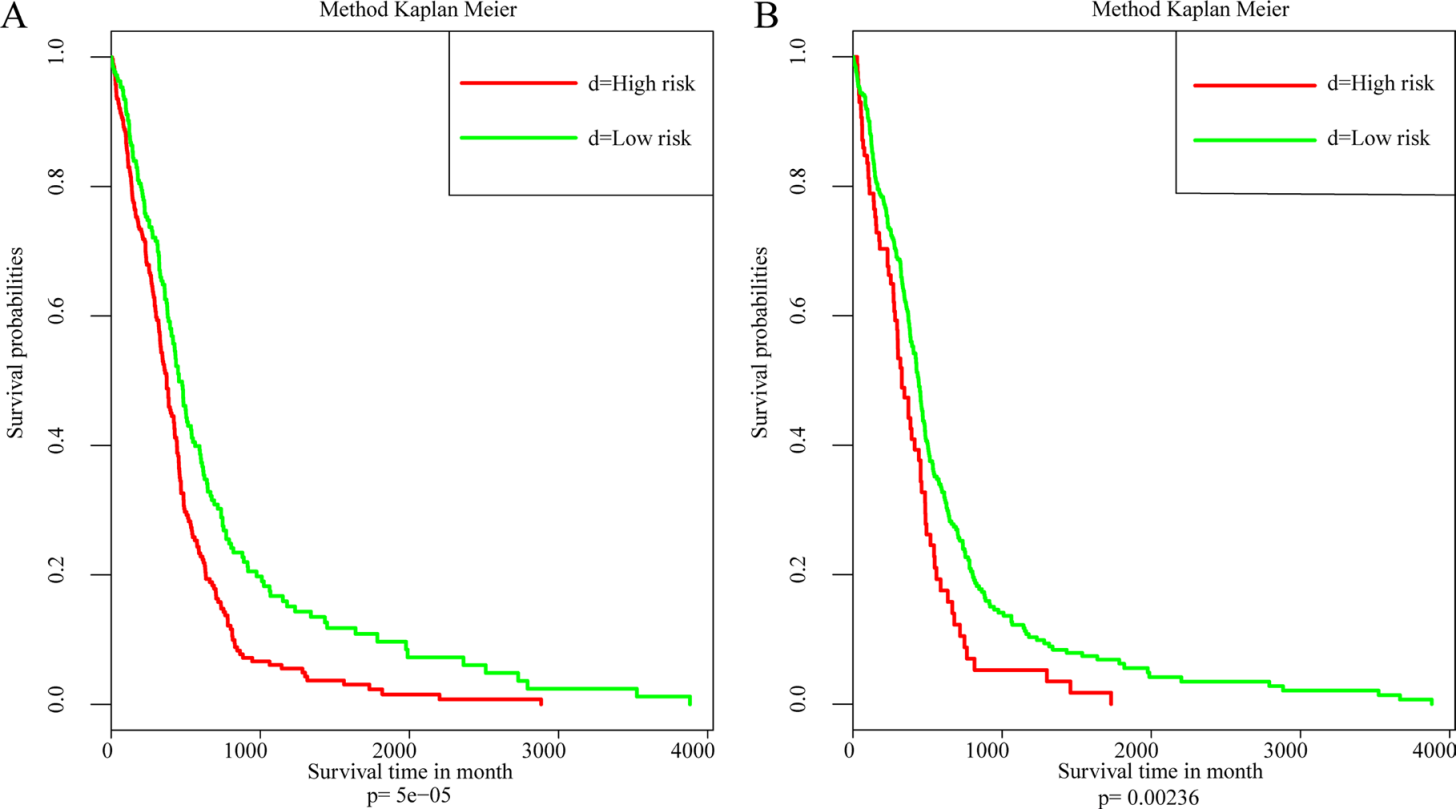
The relation between 5 driver genes and clinical characteristics (**A**) Kaplan–Meier survival analysis of samples. (**B**) Kaplan–Meier survival analysis based on external data.

Another gene expression profile from The Cancer Genome Atlas (TCGA) database [8] was utilized for external data validation which was based on the AgilentG4502A_07_2 array. A total of 483 patient samples and 466 with their clinical follow-up information were available. The results of COX multivariate survival analysis are shown in Figure 6A. It can be seen from the data that the influence of driver genes on prognosis were verified Figure 6B.

## DISCUSSION

Glioblastoma multiforme (GBM) is a common brain cancer with a dismal prognosis [1]. Although a lot of effort has been focused on increasing the GBM survival rate in recent years, the mortality rate remains high. Traditional standard therapy, which involved surgical resection and adjuvant radiation therapy, was insufficient for predicting the risk of tumor recurrence and satellite tumor transfer [2, 3]. With the development of microarray technology, increasing data resources regarding DNA repair genes are available for comparing and analyzing in the cancer field. For this particular study, the purpose was to obtain the gene expression profile of DNA repair genes across different molecular subtypes, then explore the potential relation between prognostic value and expression patterns of DNA repair genes.

To be more specific, bio-information of GBM, obtained from 539 glioblastoma tumor patients in TCGA, was analyzed to determine the molecular characteristics of genes that were associated with prognosis value. In this study, a total of 15 key genes were determined through analyzing the expression level of DNA repair genes, based on gene expression profiles and, prognosis differences of GBM patients were compared to the expression patterns of four different subtypes. It can be seen from the results that the survival times of patients are significantly affected by the expression level of these DNA repair genes [5]. To be more specific, increasing DNA repair processes in cancer cells would lead to multi-drug resistance [12], which would result in a dismal prognosis.

A total of four molecular subtypes of GBM were screened by unsupervised clustering and each of them was shown to have different gene expression levels. The results indicated that these genes tend to play independent roles in biological pathways, because the overall correlation of these 15 genes was small. Compared to the correlation of individual gene in different subtypes, it can be seen that the correlation of several genes changed significantly among the four subtypes. In cluster 2, the correlation coefficient (MDC1, FAH) was −0.65 while in cluster 4, the value was 0.07. These examples indicated that the same genes generate different regulated functions in different situations which need to be further explored by literature mining.

Cluster 4 was particularly different compared with the other three clusters which had shown better prognosis. Further analysis indicated that the age distribution in cluster 4 was significantly different to the other three clusters, especially cluster 3. This indicates that age may be a key factor in relation to good prognosis.

In order to identify the expression difference between cluster 4 and the other three clusters, the correlation coefficient of cluster 4 was compared to the correlation coefficient matrix of the other three clusters. Comparing cluster 3 and cluster 4, CDK7 and FAH were significantly positive correlated in cluster 3 but negatively correlated in cluster 4. The alteration of the expression model of DNA repair genes could be the reason for different clinical survival rates between cluster 4 and other 3 clusters. Among them, CDK7, DDB2, RNH1, RFC2 and, FAH could be the potential driver genes.

From literature mining, it can be seen that the expression capacity of 5 driver genes were revealed to be a potential predictor of prognosis [12, 13]. Up-regulation of CDK7 in gastric cancer cells has been shown to promote tumor cell proliferation and predicts poor prognosis [14]. It has been reported as a typical treatment target in standard cancer therapy and showed significant function in different cancers [14–16]. DDB2 has been considered a tumor suppressor and contributes to favorable treatment outcome [13]. DDB2 was found to be involved in interstrand cross-link repair which is associated with good prognosis in GBM. In contract, in bad prognosis groups, DDB2 has showed low expression [17]. Histone deacetylase inhibitor (HDACi) therapy is a promising anticancer epigenetic treatment which is regulated by RNH1. RNH1 is reported to be a regulator of HDACi resistance, inducing drug resistance in gastric cancer [18].

The function enrichment analysis showed that driver genes were significantly enriched in DNA repair biological pathways, including global genome, nucleotide-excision repair, transcription-coupled nucleotide-excision repair, nucleic acid phosphodiester bond hydrolysis and organic cyclic compound catabolic processes. These pathways suggested that DNA repair functions are vital in the response to prognostic value. Understanding the different expression patterns of DNA repair genes in normal tissues and cancer tissue could guide the diagnosis and treatment, as well as further predict the prognosis.

In summary, molecular characterization of GBM screened from a DNA repair gene expression database shows the potential of predicting the prognosis and selecting individual targeted therapeutic approaches for different molecular subtypes of GBM. This approach could make the clinical diagnosis of GBM cheaper, more precise and, faster. However, the results required further validation with an increased number of patient samples.

## MATERIALS AND METHODS

### Data download and preprocessing

The gene expression profiles were obtained from The Cancer Genome Atlas (TCGA) database [8] which was based on AffyU133a array. A total of 539 glioblastoma tumor patient samples were selected, which included 498 primary glioblastoma patient samples. The Kyoto Encyclopedia of Genes and Genomes (KEGG, http://www.genome.jp/kegg/) database [19] was utilized for the identification of initial DNA repair genes. A total of 248 DNA repair genes were obtained from KEGG and 181 DNA repair genes were obtained from previous articles. The level 3 data was downloaded from TCGA database [8] and converted into expression measures. Afterwards, background correction and quartile data normalization were performed.

### Screening of potentially changed DNA repair genes

For the same type of disease, different patients may have a different prognosis. These may be related to different levels of gene expression. First, genes with obvious expression alterations were screened. Then, the variance and median of the expression level of these genes were calculated in all selected samples. Genes which had above 20% of the genetic variance and the top 20% genetic median were selected from database.

### Screening of DNA repair genes which related to prognosis

The survival time of patients is affected by the expression level of DNA repair genes [5]. Potentially altered genes, which were screened from patient samples, were separately analyzed using single factor survival analysis through R survival software [20]. Genes with *P*-values less than 0.05 and variance greater than 0.7 were selected.

### Glioma molecular subtype construction based on DNA repair genes

DNA repair capacity plays an important role in maintaining genetic stability and steady state of cells. Accumulated DNA repair in cancer cells would allow them to develop multi-drug resistance [12]. Glioma molecular subtypes were built by unsupervised clustering of DNA repair genes which affect prognosis.

### Clinical characteristics analysis of molecular subtypes

The Kaplan-Meier [9] single factor survival analysis was used for clinical characteristics analysis of molecular subtypes, including age, survival time and gene expression level. Different clinical characteristics were identified and compared through observing individual subtypes.

### Screening of driver genes which associated with differential clinical features among molecular subtypes

Driver genes of different molecular subtypes were screened which highlighted differences in clinical features. The expression correlation between these molecular subtypes and distribution was analyzed by Pearson Correlation Coefficient [10] and those gene sets which had significantly changed correlation between subtypes were selected as potential driver genes.

### Functional enrichment analysis of drive genes in different subtypes

Functional enrichment analysis of driver genes from subtypes was performed through R package, clusterProfiler [11], Biological pathways and biological functions were selected which involved driver genes.

### Individual prognostic factor exploring based on multifactor COX proportional hazards model

Multifactor COX proportional hazards model was built to observe the influence of driver genes on prognosis by using R survival package [20].

### Verifying clinical significance of drive genes through external data

Another gene expression profile from The Cancer Genome Atlas (TCGA) database [8] was utilized for external data validation, which was based on the AgilentG4502A_07_2 array. A total of 483 patient samples and their clinical follow-up information were available. The influence of driver genes on prognosis was verified.

## References

[1] Louis DN, Ohgaki H, Wiestler OD, Cavenee WK, Burger PC, Jouvet A, Scheithauer BW, Kleihues P (2007). The 2007 WHO classification of tumours of the central nervous system. Acta Neuropathol.

[2] Parsons DW, Jones S, Zhang X, Lin JC, Leary RJ, Angenendt P, Mankoo P, Carter H, Siu IM, Gallia GL, Olivi A, McLendon R, Rasheed BA (2008). An integrated genomic analysis of human glioblastoma multiforme. Science.

[3] Stupp R, Mason WP, van den Bent MJ, Weller M, Fisher B, Taphoorn MJ, Belanger K, Brandes AA, Marosi C, Bogdahn U, Curschmann J, Janzer RC, Ludwin SK, European Organisation for Research and Treatment of Cancer Brain Tumor and Radiotherapy Groups, and National Cancer Institute of Canada Clinical Trials Group (2005). Radiotherapy plus concomitant and adjuvant temozolomide for glioblastoma. N Engl J Med.

[4] Wood RD, Mitchell M, Sgouros J, Lindahl T (2001). Human DNA repair genes. Science.

[5] Dizdaroglu M (2015). Oxidatively induced DNA damage and its repair in cancer. Mutat Res Rev Mutat Res.

[6] Bosken CH, Wei Q, Amos CI, Spitz MR (2002). An analysis of DNA repair as a determinant of survival in patients with non-small-cell lung cancer. J Natl Cancer Inst.

[7] Reuss D, von Deimling A, von Deimling A (2009). Hereditary Tumor Syndromes and Gliomas. Gliomas.

[8] Deng M, Brägelmann J, Schultze JL, Perner S (2016). Web-TCGA: an online platform for integrated analysis of molecular cancer data sets. BMC Bioinformatics.

[9] Goel MK, Khanna P, Kishore J (2010). Understanding survival analysis: Kaplan-Meier estimate. Int J Ayurveda Res.

[10] Sedgwick P (2012). Pearson's correlation coefficient. BMJ.

[11] Yu G, Wang LG, Han Y, He QY (2012). clusterProfiler: an R package for comparing biological themes among gene clusters. OMICS.

[12] Rosell R, Lord RV, Taron M, Reguart N (2002). DNA repair and cisplatin resistance in non-small-cell lung cancer. Lung Cancer.

[13] Zhao R, Cui T, Han C, Zhang X, He J, Srivastava AK, Yu J, Wani AA, Wang QE (2015). DDB2 modulates TGF-β signal transduction in human ovarian cancer cells by downregulating NEDD4L. Nucleic Acids Res.

[14] Patel H, Abduljabbar R, Lai CF, Periyasamy M, Harrod A, Gemma C, Steel JH, Patel N, Busonero C, Jerjees D, Remenyi J, Smith S, Gomm JJ (2016). Expression of CDK7, Cyclin H, and MAT1 Is Elevated in Breast Cancer and Is Prognostic in Estrogen Receptor-Positive Breast Cancer. Clin Cancer Res.

[15] Wang Q, Li M, Zhang X, Huang H, Huang J, Ke J, Ding H, Xiao J, Shan X, Liu Q, Bao B, Yang L (2016). Upregulation of CDK7 in gastric cancer cell promotes tumor cell proliferation and predicts poor prognosis. Exp Mol Pathol.

[16] Abaza MS, Orabi KY, Al-Quattan E, Al-Attiyah RJ (2015). Growth inhibitory and chemo-sensitization effects of naringenin, a natural flavanone purified from Thymus vulgaris, on human breast and colorectal cancer. Cancer Cell Int.

[17] Batista LF, Roos WP, Christmann M, Menck CF, Kaina B (2007). Differential sensitivity of malignant glioma cells to methylating and chloroethylating anticancer drugs: p53 determines the switch by regulating xpc, ddb2, and DNA double-strand breaks. Cancer Res.

[18] Zhu Y, Das K, Wu J, Lee MH, Tan P (2014). RNH1 regulation of reactive oxygen species contributes to histone deacetylase inhibitor resistance in gastric cancer cells. Oncogene.

[19] Kanehisa M, Goto S, Sato Y, Furumichi M, Tanabe M (2012). KEGG for integration and interpretation of large-scale molecular data sets. Nucleic Acids Res.

[20] O’Quigley J, Moreau T (1986). Cox's regression model: computing a goodness of fit statistic. Comput Methods Programs Biomed.

